# Time-constrained mother and expanding market: emerging model of under-nutrition in India

**DOI:** 10.1186/s12889-016-3189-4

**Published:** 2016-07-25

**Authors:** S. Chaturvedi, S. Ramji, N. K. Arora, S. Rewal, R. Dasgupta, V. Deshmukh, for INCLEN Study Group, Vivek Adhish, Anuja Aggrawal, Harish Chellani, A. P. Dubey, Kalyan Ganguly, Kiran Goswami, K. Suresh, R. M. Pandey, M. S. Prasad, Prashant Mathur, Arti Maria, Manoja Das, Neelima Thakur, Deoki Nandan, J. P. Shivdasani, Shamim Haider, Vidya Sagar, Ashok Mishra, Praveen Gautam, Anurag Tomar, A. M. Dixit, Sanjata Choudhary, C. B. Kumar, Samendra Mohapatra, Gouri Padhya, Subhranshu Kar

**Affiliations:** 1 Department of Community Medicine, University College of Medical Sciences, Delhi, India; 2 Department of Pediatrics, Maulana Azad Medical College, New Delhi, India; 3 The INCLEN Trust International, F-1/5, Second Floor, Okhla Industrial Area, Phase-I, New Delhi, India; 4 Child Nutrition, New Delhi, India; 5 Centre of Social Medicine and Community Health, Jawaharlal Nehru University, New Delhi, India

**Keywords:** Malnutrition, Childhood under-nutrition, Care-giving, Determinants of under-nutrition, Women’s issues

## Abstract

**Background:**

Persistent high levels of under-nutrition in India despite economic growth continue to challenge political leadership and policy makers at the highest level. The present inductive enquiry was conducted to map the perceptions of mothers and other key stakeholders, to identify emerging drivers of childhood under-nutrition.

**Methods:**

We conducted a multi-centric qualitative investigation in six empowered action group states of India. The study sample included 509 in-depth interviews with mothers of undernourished and normal nourished children, policy makers, district level managers, implementer and facilitators. Sixty six focus group discussions and 72 non-formal interactions were conducted in two rounds with primary caretakers of undernourished children, Anganwadi Workers and Auxiliary Nurse Midwives.

**Results:**

Based on the perceptions of the mothers and other key stakeholders, a model evolved inductively showing core themes as drivers of under-nutrition. The most forceful emerging themes were: multitasking, time constrained mother with dwindling family support; fragile food security or seasonal food paucity; child targeted market with wide availability and consumption of ready-to-eat market food items; rising non-food expenditure, in the context of rising food prices; inadequate and inappropriate feeding; delayed recognition of under-nutrition and delayed care seeking; and inadequate responsiveness of health care system and Integrated Child Development Services (ICDS). The study emphasized that the persistence of child malnutrition in India is also tied closely to the high workload and consequent time constraint of mothers who are increasingly pursuing income generating activities and enrolled in paid labour force, without robust institutional support for childcare.

**Conclusion:**

The emerging framework needs to be further tested through mixed and multiple method research approaches to quantify the contribution of time limitation of the mother on the current burden of child under-nutrition.

**Electronic supplementary material:**

The online version of this article (doi:10.1186/s12889-016-3189-4) contains supplementary material, which is available to authorized users.

## Background

Persistence of childhood under-nutrition in India despite the economic growth and general development is a complex public health riddle that keeps challenging analysts, program managers and implementers at all levels. The modest decline of under-nutrition levels between the last two rounds of the National Family Health Survey (NFHS) is well known and India continues to remain off-track from the Millennium Development Goal (MDG-1) target [1, 2]. Prevalence of underweight children increased in eight states (Assam, Arunachal Pradesh, Bihar, Haryana, Jharkhand, Madhya Pradesh and Mizoram) between the last two rounds of NFHS, and remained stagnant in another seven states; the national level prevalence of underweight and stunting was 42 and 48 %, respectively [2]. The concern and urgency for under-nutrition was rooted in the fact that while gross domestic production (GDP) in India grew at an average of 3.95 % per capita per year between 1980 and 2005, almost half of the under five population in India continued to be undernourished [3]. The proportionate share of consumer expenditure on non-food items increased during last decade while the share of expenditure on food items especially cereals, pulses and oils decreased both in urban and rural India (66th Round of National Sample Survey, 2009–2010) [4]. With increasing household income, children from better off families and with educated mothers had greater nutritional advantages compared to those from less privileged classes [5].

## Context

Black et al. have proposed a more inclusive framework than the earlier UNICEF model to understand child malnutrition by encompassing both under-nutrition and childhood overweight in low and middle income countries [6, 7]. While the UNICEF framework outlined relationships between poverty, food insecurity and other underlying and immediate causes to under-nutrition, the new framework goes beyond determinants and focuses on actions to achieve optimal child nutrition. These frameworks are based on statistical analyses of large-scale anthropometric surveys and are essentially positivist and deductive. They would naturally be nomothetic in approach and seek to find near-universal solutions for what the Lancet Series calls ‘global nutrition system’. Narrow pragmatic explanatory models may overlook the context and the associated politics as well as miss the nuances of human suffering and adaptation [8].

Women in developing countries are rapidly entering the formal and informal job markets in addition to accomplishing their domestic responsibilities [9]. A significant proportion of Indian women in both rural and urban areas are engaged in income generating activities and employment, marked by rapid feminization of farming and labour [2, 10]. Women from households in the bottom quintiles in least developed villages are more likely to work outside their homes for extended periods of time than those in higher income scale [10]. Poverty and resource availability at household level, and mothers’ preoccupation with household and out of home activities influence the timing, quality and quantity of child’s food and childcare in general [11, 12].

Our study adopts an inductive approach using multi-centre qualitative research methods to derive contextual understanding of pathways and processes associated with under-nutrition in the context of chronic poverty settings of India. The specific objective of the study was to: (i) map maternal perceptions of processes of childhood under-nutrition, and under that broad rubric explore intersections of chronic poverty, food availability, childcare, markets and public services; and, (ii) their influence on nutritional status of under five children (two age strata: 6–24 months & 25–60 months). We recognized that decisions affecting intakes (and thereby nutritional status) are mostly made and practised at the household level – the mother being the central protagonist.

## Methods

### Study setting

We conducted this study in six districts spread across equal number of Indian states namely: Samastipur (Bihar); Gumla (Jharkhand); Sonepur (Odisha); Tikamgarh (Madhya Pradesh); Chittorgarh (Rajasthan) and Mathura (Uttar Pradesh). These states are termed as the Empowered Action Group (EAG) states due to their poor human development indicators [13]. Districts with median prevalence of under-nutrition (using weight-for-age) in the respective states using District Level Health Survey- 2 [DLHS-2], (2002–2004) data [14] were selected for the study. In every district, five villages were purposively selected for in-depth interviews (IDIs) with mothers in a manner that these villages fell under the jurisdiction of separate primary health centres and dispersed across the rural part of the district. Three urban slums were also identified in the district - one located near the centre and two at the periphery of the district headquarter. The villages and urban slums selected for focus group discussion (FGDs) with mothers and other family members were different from those identified for in-depth interviews (IDIs) of mothers. All the interviews took place at respondents’ residence, with prior consent. In addition, non-formal interactions (NFIs) were also conducted with a range of stakeholders to explore some sensitive domains of enquiry.

### Recruitment and sample selection

The profile and number of stakeholders interviewed and FGDs/NFIs conducted in each of the states/districts are illustrated in Table 1.

**Table 1:** Profile of in-depth interviews, focus group discussions and non-formal interactions

A: Stakeholders at state, district, and block levels	Total done in six states
State Level^a^	17
District Level/Block Level^b^	34
Total state, district and block levels	51
B: Stakeholders at community level	
Anganwadi Worker (AWW)	12
Auxiliary Nurse Midwife (ANM ‘S)	12
ASHA/USHA/Link worker	8
Non Government Organization (NGO)/Community Based Organization (CBO)	10
Community Leader	10
Gram Sabha/Zila Parishad	12
Self Help Group	10
Total community level	74
C: Mothers of Index Children	
Mothers of moderately undernourished children ^c^	192
Mothers of mildly undernourished children ^c^	96
Mothers of normally nourished children ^c^	96
Total mothers	384
D: First Round Focus Ggroup Discussion (FGD)	
Mothers of < 2 years	6
Mothers of 2–5 years	6
Grandmothers of < 2 years	6
Grandmothers of 2–5 years	6
Father	12
Anganwadi Workers (AWW)	12
Auxiliary Nurse Midwife (ANM)	6
Total First Round of FGD	54
E: Second Round FGDs	
Mother (Rural) of <2 years	6
Mother-in-law (Urban)	6
Total Second Round of FGD	12
F: Non-Formal Interactions (NFI)	
Mothers of <2 and 2–5 years	20
Grandmothers	8
Fathers	14
Grandfathers	8
ANM/AWW/Accredited Social Health Activist (ASHA)	18
Doctors (Public Health Center/Non formal)	4
Total Non-formal Interaction	72

^a^Director of health services(DHS), Director reproductive child health (RCH), Mission director National health mission (NHM), Director women and child development

^b^District Magistrate, Chief medical officer, Medical superintendent, District program officer, child development program officer, Integrated Child Development Services (ICDS) supervisors

^c^The weight for age reference of World Health Organization was utilized to define nutrition and its severity: Normal – SD score > −1; Mild Under-nutrition – SD score < − 1 to > − 2; Moderate Under-nutrition – SD score < −2 to > −3; Severe Under-nutrition – SD score < −3

We identified mothers of under-five children with the help of the local frontline workers (Anganwadi Workers from Integrated Child Development Services (ICDS) program). Purposive sampling was done to obtain representation from both backward (scheduled castes, scheduled tribes and other backward communities) and forward communities. FGDs and NFIs were conducted with a range of stakeholders (Table 1) but none of these had participated in the IDIs and were not directly related to the mothers who participated in the IDIs. The selection of respondents for NFIs was purposive and based on the perception of senior investigators about the individuals who would be able to provide additional and supplementary information on the emerging themes incompletely explored during the first round of data collection. During the interaction, the interviewer had conversation with the respondent without using structured interview guide although the broad framework of NFIs and the guide of the second round of FGDs were similar. Brief notes of the conversation were made either during the interaction or later (no audio or video recording). The information gathered was then added and triangulated while writing the results.

### Interview guides

IDI guides were structured and open ended. A mulit-disciplinary team developed the IDI and FGD guides covering the broad domains of: perceptions about nutritional status of children; current feeding and child care practices; availability of mother and other family members for the care of the child; socio-cultural beliefs and practices of pregnant women, lactating mothers and children; market and food for children; identification and management of under-nutrition; biological factors; growth monitoring; and policy & program environment. The guides were translated in local languages, pilot tested in all the states and the problems resolved during the national orientation workshop. We carried out FGDs in two rounds; the first round was completed along with the IDIs (Table 1). The interim analysis and interpretation indicated emergence of themes and domains that were inadequately covered in the IDIs and first set of FGDs. Hence the second round of FGDs, along with NFIs with a range of stakeholders, were conducted (Table 1) with revised guides and checklists. These were informed by emerging emphasis on issues of busy mothers, child care, coping strategies adopted by time constrained mothers, intra familial dynamics of child rearing, influence and use of “ready - to - eat” market foods, and other issues like household expenditure on non-food items, and men’s alcoholism.

We audio recorded IDIs and FGDs and written verbatim notes were later supplemented by transcripts of audio tapes, before final translation into English. Besides this, field notes were also prepared for the same.

### Data processing and analysis

The data was coded and analysed jointly by the authors (core investigators) of the manuscripts to ensure consistency in interpretation and reliability. To keep an overview of the entire data, cross-tabulation of axial and selective codes across the respondent categories and study sites was prepared. This also helped us to separately analyze data from each state by stakeholder category. Triangulation was done at two levels – across methods and across respondents. Findings from IDIs, two sets of FGDs and NFIs were compared. The findings were assessed for similarities and differences in perceptions across stakeholders. Findings from six states were conveying similar interpretation and hence further reinforced the validity of findings. In view of these observations and since all sites represented similar economic and social development status, the investigator group decided to merge the findings from all sites [15]. We adopted the grounded theory approach to develop an inductively derived explanation about the phenomena emerging from the data [16] instead of forcing or testing a priori hypothesis [17]. The evidence and theories were allowed to emerge through stepwise process of analysis [18, 19]. All the steps of analysis (open, axial and selective coding) were followed consistently to have inter-coder reliability and have stability and reproducibility for the whole data analysis. The data analysis was first done independently by the researchers followed by series of joint work sessions between the investigators and research team to arrive at a consensus list of axial and selective codes. However, the assigning of all axial codes and selective codes to every open coding response was done by three investigators (NKA, SR & VD) together thereafter approximately 10 % was re-assessed for appropriateness and consistency by the remaining members of the investigator team. In open coding, the data was fractured into small fragments [20] (free listing). During open coding, relevant/important statements or quotes were marked for use in the report as reference material. Axial codes emerged keeping in mind the connection/relationship between free listed responses/small units [21]. Still broader domains were identified by grouping the axial codes which were connected/related to each other (selective codes) [21]. The researcher team thereafter assigned axial codes to every fractured small units in the first round and selective code (wherever applicable) in the second round. The senior team members divided the FGDs and NFIs among themselves and summarized their inferences individually. Thereafter, through three day-long sessions, they arrived at consensus on the emerging themes.

### Conceptual framework and emergence of proposed model

We outlined a preliminary conceptual framework of causation of under-nutrition which was an adaptation of the widely accepted explanations and model proposed by UNICEF [22, 23]. However, as data analysis started, the emergence of newer explanation(s) was allowed to shape and redefine the framework. This was done while accommodating the “*emic*” perspective on various domains of enquiry, along with the “*etic*” perspective [24]. Concept identification began with the first set of interviews with mothers and providers and FGDs, where data collection was alternated with analysis. Initial open-coding helped in revealing the text of the interactions which was subjected to intensive scrutiny. We also identified properties and dimensions of the phenomena, giving it specificity [18]. Data were then woven around the phenomena by axial coding. Thus, the major themes began to emerge from the data. Finally in selective coding, construct of core themes emerged. We reached a point when data seemed repetitive, thus, data saturation was achieved. The model finally arrived at went beyond mere reconstruction of events, it was a co-construction between researchers and participants [25]. It was also reflective of changing ground reality and the conditions that led to these problems [20].

### Respondent validation

After the analysis of data, we went back to the same population for respondent validation of the results with another set of mothers (15–18 mothers in each group drawn from all social and economic classes). The emerging model of under-nutrition and the fittest narrative in the most likely scenario were discussed for their feedback, criticism and concurrence.

### Quality assurance measures

A multi-disciplinary team comprising of program evaluation experts, health social scientists, anthropologists, public health specialists and epidemiologists supervised and assured the quality of protocol and guides’ development; data collection; analysis and interpretation. A dedicated central coordinating team (CCT), lead by authors, supervised the data collection, data transmission, data management and analysis. Site investigators and field teams were oriented to study protocol and provided hands-on practice for doing IDIs before the launch of the study. Oversight of the field data collection was provided by the site investigators and CCT members made site visits in the initial phase of the study to get first hand understanding of the quality of data collection and took steps to improve the performance of field teams if required. CCT members conducted all the FGDs (both first and second round) and NFIs. In the present study, 100 % data were re-checked at the Central coordinating office (CCO) for quality of translation, transcription and thereafter closely explored for issues and themes which were critical for developing a new conceptual framework of under-nutrition in children. Steps were taken to assure consistency in coding during entire process of data analysis [26]. Due to inherent limitations of interpretation of the qualitative data from different parts of the country, CCT had a series of meetings to discuss the data and its contextual interpretation. Respondent validation was also a measure of quality assurance for the inferences drawn from the emerging evidence.

## Results

A total of 509 IDIs, 66 FGDs and 72 NFIs were held in six districts across same number of Indian states (Table 1). Furthermore, seven respondent validation exercises were conducted with mothers (at study sites and an additional site in Palwal, Haryana).

### Emerging model of child under-nutrition based on the perceptions of the mothers and the key stakeholders

**Fig. 1:**
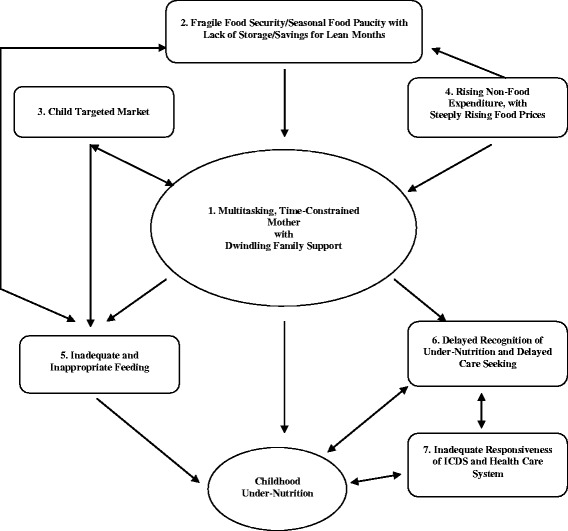
Emerging model of childhood under-nutrition. The figure is a graphic representation of the core themes and sub themes that became apparent from the data, dynamics of relationships between these and their association with childhood under-nutrition

**Fig. 2:**
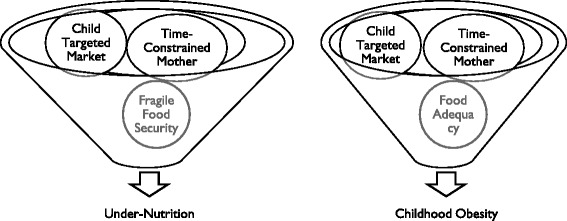
Potential of both childhood under-nutrition and obesity (Double Burden) with changing economic condition and food security at household level: The figure depicts the possible scenarios of double burden and illustrates that the themes of time constrained mother, child targeted market, and food security can lead to dissimilar outcomes in different settings

A construct evolved showing core themes and associated sub themes as components of the model with the dynamic relationship among the components as the process. The respondent validation refined and finalised the model and helped in reaching the fittest narrative (Fig. 1). The most forceful and substantive core themes emerging as the drivers of under-nutrition were: multitasking, time constrained mothers with dwindling family support (core theme 1), and child targeted market for ‘ready-to-eat’ food items (core theme 3) in the background of widespread poverty and fragile food security (core theme 2). Modifications made during the process of respondent validation were: incorporation of “lack of storage/saving for lean months” and “lack of capacity for bulk purchase” in the core theme of “fragile food security and seasonal food paucity” (core theme 2); highlighting the share of “alcohol” within the core theme of “rising non-food expenditure” (core theme 4) and adding a backdrop of “culture-bound/chronic deprivation” to the core theme of “Inadequate and inappropriate home feeding” (core theme 5). Participants of respondent validation exercise also came up with the possible circumstances when issues related to poverty and fragile food security may not be present any more at household level but mothers shall continue to remain time constrained (core theme 1) because of their pre occupation with professional and service responsibilities outside homes. In such scenario mothers shall continue to explore options that are time sparing, child targeted marketing of “ready-to-eat” snacks and foods (core theme 3) might become even more aggressive, be able to penetrate to the geographical and social peripheries and, childhood obesity may also emerge as part of the nutrition transition in our society leading to double burden (Fig. 2).

### Description of core themes

The core themes that emerged through the data are outlined in following paragraphs. (Detailed list of themes and perceptions observed during analysis are provided in the Additional file 1: Table S1).

#### Core theme 1: Multitasking, time constrained mother with dwindling family support

The participating mothers perceived that they were busy with several daily tasks and activities that went beyond routine household work. They were involved in, one or more of the tasks outside home related to: farming, farm labour, cattle rearing, and poultry related work in addition to household work. A large number of women were working in the Mahatma Gandhi National Rural Employment Guarantee Act (MNREGA) [27]. To supplement family income, mothers perceived that they remained outside their homes for eight hours or more and were able to spend only one to three hours with their under-five children.

There were also several mothers who spent less than an hour daily for child caring. Mothers of undernourished children were able to spend lesser time for child caring as compared to their counterparts who had better nourished children. The issues surrounding time constraint for mothers appeared to amplify further in the context of nuclear families, declining support from elders and other family members and minimal participation of father in child’s food preparation, offering and feeding. Mothers felt that these circumstances left little time with them for their own care too.

Mothers frequently entrusted the care of their younger child to an elder sibling, especially a girl. Elder siblings were not preferred to look after children <24 months, if there were elder family members or neighbours available, or if the elder sibling was attending a school. The data suggested that mothers had reasonably good knowledge about how to keep a child healthy through "good food" and "good care", in their local context. Homemade food was perceived as ‘good’ by the mothers and perceived that energy dense and high protein containing food items as desirable and healthy for their young children. These included examples like putting ghee (clarified animal fat) in all weaning foods, halwa (sweet semolina with sugar and oil), *kheer* (rice-milk pudding), dry fruits, eggs, chicken and fish. It was interesting to note that many of them also perceived store purchased fast food items and food supplements like Horlicks acceptable and ‘good’ food that can be given to children. The mothers viewed taking care of personal hygiene, sanitation, offering good foods in a timely and correct fashion as indicators of ‘good quality’ of the care for a child. Other components of good care of a child were physical expression of love and affection, timely care seeking for illness and education. Mothers from urban slums appeared to perceive greater role of good food given in a timely manner for a healthy child as compared to their rural counterparts. However, these mothers cited their preoccupation with various activities inside and outside the house besides poverty and resource constraint within their households, as a vital factor for failing to put their knowledge into practice in a routine manner. The study was not designed to address differences of perceptions between social strata.

…I get busy with other household work. So, I do not have time for my child because of overload of work. [IDI, Mother, Madhya Pradesh].

I cook food when I come back from work, do other household work - so the day is over and I am unable to spend any time with her [the child]. [IDI, Mother, Bihar]

We go to work outside home so unable to give proper care to children. Income is more important for us. [IDI, Mother, Rajasthan]

They (the mothers) go out to work, leaving all the responsibility on the siblings for feeding the children. [FGD, ASHAs, Rajasthan]

#### Core theme 2: Fragile food security/seasonal food paucity

This was an overwhelmingly compelling theme. The evidence from the six states indicated that almost all study households experienced some type of food shortage, including seasonal shortages. Even the “not so poor” households faced seasonal paucity of foods. The principal factors were: chronic poverty; lack of storage facility at household; lack of enough savings for lean months; and lack of capacity for bulk purchases, in the wake of steeply rising food prices. Although, the households experiencing fragile food security gave preference to younger children during the intra-familial distribution of food and milk, their food intake remained inadequate in both, quantity and quality through most periods of the year due to underlying poverty.

Being a daily wage earner I only earn rupees 50 only. Most of it is spent on medicines. I am without work for five days. How can I give her pulse and ghee. [IDI, Mother, Bihar]

The child remains well till s/he takes mother’s milk. But, after 6 months when the amount of mother’s milk is reduced and child needs semi-solid food, it is not available in home. Only rice is available. Even so, mothers have no time to feed that rice to children. Fathers are drunk most of the time, so they are unable to take care of children; then the child slides into under-nutrition. [IDI, Medical Officer, Jharkhand]

When the food is less in the household, children get affected the most. When we have less rice, we eat small amounts. In such situations, we feed our children first; then we take rice with salt. It gets worse many a times. [FGD, Fathers, Jharkhand]

#### Core theme 3: Child targeted market with wide availability and consumption of ready-to-eat market food items

The mothers of both age groups, namely 6–24 months and 25–60 months old, regularly spent money to purchase “ready - to - eat” snacks/fast foods and drinks. These included traditional Indian snacks prepared by local vendors and sweetmeat shops. It was interesting to note that commercially packaged snacks were available in small affordable packs and portions across the study sites; some villages were remote and had no motor-able roads. Most of the stakeholders from all the categories including mothers felt that children readily accepted these because of colour, flavour, taste and variety, were often part of daily menu for several young children and frequently replaced regular home meals. Mothers perceived these items as a solution to their own time constraints although homemade food was preferred and considered “good food”.

Several doctors and frontline health workers perceived as the immediate role models for the rural and slum dwelling mothers, were noticed by the mothers giving commercially available food supplements/weaning substitutes and ‘health drinks’ to their children and occasionally also prescribed for their patients.

Biscuits, ice-cream, *Kurkure*, Maggi (instant noodles), she [the child] eats [these snacks] 4–5 times a week. When she takes these snacks, she is unable to eat homemade foods and skip lunch. [FGD, Mother, Rajasthan]

If the family is economically not well off, even then the child gets Rupee 1 daily. At home, snacks like *mathari* (made of flour and oil) are to be prepared and kept for the child to munch. The child still throws tantrums, is hell bent on going and getting snacks from the shop. [NFI, Mother in Law, Madhya Pradesh]

Parents don’t have time in the morning to prepare school tiffin box. They find it easier to put a pack of crisps or some other snacks from the market in the school bag. [NFI, ANM, Jharkhand]

Young mothers are not like us. They are lazy. They will prefer giving two rupees to child instead of packing rice for school. [FGD, Mothers-in-law, Jharkhand]

I am giving *Horlicks* [health drink] to my son since he was very young because *Horlicks* provides strength. Mothers prefer to give *Horlicks* to their children after watching TV advertisements. [Doctor, echoed by several ANMs and AWWs, Jharkhand]

We go for work leaving them in the house. If on our return we find that they have not eaten we get some eatables from nearby shops for the children as it is already late. [FGD, Father, Jharkhand]

#### Core theme 4: Rising non-food expenditure along with rising food prices

There was widespread impression among mothers and other community stakeholders that household incomes had increased during last few years. Medical costs, household goods like furniture, cycle, alcohol, education, travel, communication (mobile phone), clothing, and some savings, if possible, were the major heads for rising ‘non-food’ expenditure. Alcohol emerged as an important expenditure with rising household incomes in many communities, especially the tribal areas; this restricted the availability of money for other expenditures. Recent rise in food prices and overall high inflation rates were felt as additional factors that adversely influenced household food availability and consumption despite increase in the absolute incomes.

When I have money available at home, I usually spent on household items like cloths, cycle, TV, mobile and sometimes it is spent on medical care, market foods for children. [NFI, Mother, Uttar Pradesh]

If the husband is a drunkard, then mother is not able to give attention to the child. Sometimes the child is sick and does not get proper attention. [IDI, Mother, Bihar]

#### Core theme 5: Inadequate and inappropriate feeding

Non-exclusive breast feeding in first six months; delayed complementary feeding; gender preference (preferring the boy child); reduced food during sickness; low energy and nutrient density of weaning foods; lack of special efforts to modify and or customise food for the child; and dilution of milk were main fault lines in the feeding practices that kept emerging through the data. Many of the children in the age group of 6–24 months were being breast fed, but very few had been exclusively breast fed till 6 months of age. Data suggested that complimentary feeding was started either too early (within first three months of life) or too late (after one year) and the foods lacked both in quantity and quality (i.e. nutrient and energy density). Mothers felt that they knew what is good for the child but did not have many options, time and resources. The narrative that emerged through the data was indicative that at least some of the inadequate and inappropriate feeding practices could have also resulted from the lack of awareness; long standing habits and culture bound traditions could also not be ruled out for several of the prevalent infant feeding practices.

We are poor people, from where will I get milk. If we add water, then the child won’t suffer from loose motion. Milk leads to constipation we also remove cream from milk because it will stick in the child mouth. [F G D, Mother, Bihar]

Semi-solid food is not given to the child at six months as it may cause weakening of the limbs. Older persons of family say that there might be pooling of cereals in the child’s stomach. [IDI, Mother, Madhya Pradesh]

I stop feeding *roti* (indigenous bread) during diarrhoea and give tea or *lassi* (curd drink with sugar). [IDI, Mother, Rajasthan]

(During illness) I stopped giving solid food and cow’s milk may be difficult to digest, so I stopped giving other foods. I only gave *dal* [lentil] water. [IDI, Mother, Jharkhand]

Mothers do not want to breastfeed their children and they (children) remain hungry. If mother’s milk is given, mother becomes weak and her shape is distorted. [F G D, Mother in Law, Rajasthan]

#### Core theme 6: Delayed recognition of under-nutrition and delayed care seeking

Several mothers had wrong perception about the nutritional status of their children and misclassified them. They believed that that the physical appearance, irritability and repeated illness of the children were good guides about their nutritional status but only a few mentioned anthropometries to recognize under-nutrition. Providers at the grass root level viz. ANM, AWW and ASHA perceived callous attitude and busy family members including mothers as the key reasons for frequent delay at household level in seeking professional help for their sick and undernourished children. On the contrary, mothers said that they seek care as soon as they feel the child required a provider’s opinion and only a few attributed the delay occurring if any to their pre occupation with other activities.

I can identify undernourished child by looking at its face. [IDI, Mother, Rajasthan]

He [the child] doesn’t play, he never goes away from the mother, looks weak, not looking good to the eyes, they became lean, their face,ears, legs, hand became dry.” [IDI, Mother, Orissa]

If we became careless, then child suffers from cold. After that, most of the families give homemade medicines and other medicines available at home to child before taking him/her [the child] to hospital. [F G D, Father, Madhya Pradesh]

In village, people go to field and do not have time for children. They think we will go to health facility next morning, and again in morning they do not have time next day either. [IDI, ANM, Rajasthan]

#### Core theme 7: Inadequate responsiveness of ICDS (Integrated Child Development Scheme) and health care system

The mothers were aware of the ICDS, its activities in their area (Anganwadi Centres - AWCs), and regarded supplementary feeding as its main activity but were unsatisfied with their functioning.

The general perception among the community about the ICDS and health systems was: problems in the delivery of supplementary nutrition; poor quality of services and environment at AWCs; no sustained efforts for recognition of nutritional status of children and their subsequent management at either ICDS centres or health facilities; missed opportunities for nutritional assessment, education and rehabilitation while delivering curative services.

No separate management unit for under-nutrition is available at the community health center (Community Health Center). [IDI, District Level Officer, Rajasthan]

Those children who are undernourished will take 5 days in their recovery instead of 3 days but we cannot allow overstay to them on this ground; we send them by giving proper advice. [IDI, Medical Officer, Bihar]

We do not have faith in government health facilities. There is always shortage of medicine too. In private clinics, the rush is less and the doctor is able to pay adequate attention [IDI, Mother, UP]

She (Anganwadi Worker –AWW0 does not take weight of child. She cooks food in her house and keeps the children waiting outside. [F G D, Mother, Bihar]

AWW-Helper cooks rice for children (and makes them wait). During this period, children fight with each other and come back home crying. Once, he [the child] got his hand fractured. AWW often says that she is going for some meeting. [IDI, Mother, Jharkhand]

## Discussion

The inductively derived model (Fig. 1) from the data collected in the study is based on perceptions of the mothers and the other key stakeholders. The study states/districts were remarkable for some of the worst nutritional indicators including stunting, and hunger indices in the alarming category [28] and lowest ranks in the matter of Multi-dimensional Poverty Index (MPI) [29]. The data did not detect any interstate differences and in fact reflected consistency of the findings based on both primary data and subsequently respondent validation. It was therefore decided to present the results in combined form from all the six study sites. The most forceful and substantive core themes emerging as the drivers of under-nutrition were: multitasking, time constrained mothers with dwindling family support (core theme 1), and child targeted market (core theme 3) which facilitated almost universal availability of “ready-to-eat” market foods in the background of widespread poverty and fragile food security (core theme 2). The proposed framework alone cannot, and does not, attempt to comprehensively explain its components and pathways in the causation of under-nutrition.

The key finding across the study areas is one of overwhelming vulnerability; variously described in poverty literature as unpredictability and riskiness found in the lives of the poor [30] or, the threat of poverty [31]. Behaviours like diluting milk, and weaning with household foods meant for adults (instead of specially prepared semi-solid foods) could all be traced to chronic poverty and long standing deprivations thus marking a transition from reasoned choice/compromise to the realm of cultural wisdom [32]. Chronic poverty literature is increasingly taking up the challenge of understanding how much poverty is chronic that would be expected to persist into the future [33].

Work load and time constraint of the mothers emerged in the present study as the most forceful component contributing to child under-nutrition. Engle and Menon [34] report expanded on the UNICEF model of nutrition to include care and associated resources as important elements for creating an environment in which children remain adequately nourished and healthy. Mothers in the present study perceived that they spent eight or more hours working outside home, and spent less than three hours per day attending to the needs of their young children; mothers of malnourished children were spending even lesser time with their young ones. Mothers seemed helpless due to limited time availability to prepare, customise, and feed their children that is appropriate and adequate despite increase in family incomes and their perceived understanding of the ‘good food’ and ‘good care’ for the child. They were looking for solutions. According to literature, working mothers are able to spend much less time with their young children [35]. In a study from South India, working mothers on an average spent less than two hours daily with their children whereas non working women spent three-four hours per day [36]. Iranian women from the lowest economic strata worked harder to make up for the family incomes but their young children (less than 2.5 years) continued to consume lower energy and were thinner than children of women from higher income group and doing light work [35]. In another study from Portugal, factors like adverse familial structure, hospitalization, mother’s mental health, family stress, factors including alcoholism and child’s age when mother resumed her job influenced mother’s ability to look after her children and their nutritional status [37]. Researchers from Nepal reported that peer child care in households with non working mothers was not associated with malnutrition, but became a significant risk factor among children of working mothers [38]. Data from Indonesia suggested that mothers and care givers need support and adequate resources to perform child care activities irrespective of child’s nutritional status or mother’s employment status [39]. We found that most of the mothers across the six states had little or no help from their husbands and elders in the family and had to seek help from offspring for the care of their younger sibs. Issues of gender and patriarchy operate within this framework. Researchers from other parts of India have also reported that paternal involvement in care of preschool children is usually minimal and does not vary with maternal employment or family structure [40]. The dilemma between earning and caring has implications for breast feeding too.

The breast feeding rates continue to remain at unacceptably low levels in India [2, 41] and this is in consonance with analyses of large scale survey data from other developing countries as well [42, 43]. The data from the current study suggested that weaning either occurred too early (≤3 months) or very late (beyond the age of one year) and this may have resulted from several factors including early resumption of her outside home economic activities along with minimal support within the family environment.

Time constraint for the main care provider at home – the mother – has created an opportunity for “ready-to-eat” snacks and market foods, to gain entry into marginalised households as well, often replacing freshly cooked home foods. The emerging popularity of such foods has been attributed to ready availability, taste, low cost, marketing strategies and peer pressure [44]. Research in recent years has shown that ‘social norming’ effects like aspirations and social mobility are particularly powerful and seem to legitimise the buying and consumption of store bought foods [45]. Marketers recognize the particular vulnerability of children to marketing and advertising. Public health experts have expressed concern about irresistible attraction and wide availability of processed, “ready-to-eat” food items which have been incriminated as important drivers of the double burden of under and over-nutrition [46, 47]. Changes in the children’s food consumption behaviour in the context of busy mothers could also allow for the potential deficiency of nutrients caused by the increased intake of fast foods and snacks [48, 49]. Although our mothers perceived home cooked food ‘good’, but children wanted to have these “ready-to-eat” food items purchased from the stores and the mother perceived relief from severe time constraint for herself in the background of their wide availability. The overall nutritional impact of these “ready-to-eat” snacks and food items on the study population was not assessed by us but by and large store purchased food items are processed, high in salt, sugar and fat and in most instances have limited nutritional value [45]. In a recent systematic review of literature from Asia, the authors have explained the widespread existence of double burden during nutrition transition due to variable co-existence of situations of food security with plenty and poverty at household level [50]. An important perception and associated framework that emerged during ‘client validation’ exercise of the study was the possibility of childhood obesity occurring concurrently with under-nutrition (double burden) when concerns of fragile food security at household level reduced and mothers continue to remain time constrained (Fig. 2). Food and beverage industry is now targeting low and middle income countries and have been able to penetrate the local food systems extensively [51]. Weak regulatory and implementation frameworks render communities particularly those with weaker purchasing powers and information networks vulnerable to additional nutritional risks [52]. Men’s alcoholism is an emerging yet under represented cause of chronic under -nutrition among women and children. To an extent, this is happening in developed parts of the world as well [53].

This study explores in depth the child feeding and nutrition issues from the perspectives of mothers supplemented with that from several key stakeholders in chronic poverty settings in India. The current child feeding behavior and nutritional status might be combined influence of several factors including poverty, time constraint of the mother, child targeted marketing and mothers’ ignorance; some of this might be belief driven or culture bound. The data does not quantify the contribution of various contributing factors. Therefore, there is need to undertake mixed and multi-method studies to measure the influence mother’s time constraint has on: quality and quantity of food intake by the young children, consumption of ‘ready-to-eat’ store purchased foods by children, women’s awareness about appropriate and adequate feeding practices, timely care seeking during illness and responsiveness of public sector services provided by ICDS and health department in perpetuating child under-nutrition in the context of fragile food security. Equally important agenda for the poor but rapidly developing communities now is to pursue organisational research on work-family conciliation with clear delineation of roles for different actors in mothers’ environment, including the state [54]. The study was undertaken in some of the poorest regions of India and these findings need to be interpreted accordingly.

## Conclusion

Our qualitative data does reaffirm that programmes aimed at correcting severe chronic malnutrition require well-rounded multi-sectoral approaches that should give high priority to address challenges of mothers time limitations while sustaining means of their livelihood through innovative and locally feasible models [55, 56]. To carve out pragmatic roadmaps, communities need to consider: (i) *Whole of Government approach* – inter-sectoral coordination for policy and program development at national and state levels; and (ii) *Whole of Society approach* – harmonization of actions at the point of implementation for convergence of economics, nutrition and health [57]. Several recent grassroots initiatives in the country including the study states are seeking to provide institutional support to young children of working mothers with the provisions of crèches/day care centres (ICDS-IV) [58, 59] and LPG connections to poor households. Such community based responses are encouraging, likely to empower the busy mothers and may signal a significant shift in effectively tackling childhood under–nutrition.

## Abbreviations

ANM, Auxiliary Nurse Midwife; ASHA, Accredited Social Health Activist; AWW, Anganwadi Worker; CHC, Community Health Center; DHO, District Health Officer; DHS, Director of Health Services; DLHS, District Level Health Survey; DM, District Magistrate; EAG, Empowered Action Group; FGD, Focus Group Discussion; GOI, Government of India; ICDS, Integrated Child Development Services; IDI, in-depth interviews; INCLEN, International Clinical Epidemiology Network; India-CLEN, Indian Clinical Epidemiology Network; MNREGA, Mahatma Gandhi National Rural Employment Guarantee Act; MoHFW, Ministry of Health and Family Welfare; MPI, Multi-dimensional Poverty Index; MWCD, Ministry of Women and Child Development; NFHS, National Family Health Survey; NFI, non-formal interactions; NGO, Non-Governmental Organization; PHC, Primary Health Centre; PI, Principal Investigator; RA, Research Assistant; U-5 MR, Under-Five Mortality Rate

## Additional file

Additional file 1: Table S1. Detailed list of themes and perceptions observed during analysis. (DOC 45 kb)
