# Supplementary material for: Time-constrained mother and expanding market: emerging model of under-nutrition in India
Source: BMC Public Health. 2016 Jul 25;16:632. doi: 10.1186/s12889-016-3189-4 (PMC4960674; doi:10.1186/s12889-016-3189-4)
Supplement: Additional file 1: Table S1. — Detailed list of themes and perceptions observed during analysis. (DOC 45 kb) [file 12889_2016_3189_MOESM1_ESM.doc]

| **Additional file 1: Table S1: Detailed list of themes and perceptions observed during analysis** |
| --- |
| **Emerging Themes and Underlying Factors of Child Under-nutrition**   - **Time-constrained mother with dwindling family support:** busy mother and poor child rearing practices. - **Poverty/food security** - **Inadequate and inappropriate feeding:** faulty feeding habits, delayed weaning, food with low energy density, low nutrition density, less quantity, dal/rice water. - **Child target market foods:** traditional locally made snacks, halwai sweets, maggie, noodles, kurkure, burger, biscuit, toffee, chips, cheap local snacks imitation of established brands. - **Spending pattern:** use of family income for household items, furniture, mobile phone, two-wheelers, alcohol, wine, tobacco, fast food. - **Quality of and access to health facility:** quality of and access to health facility, reach of program, focus on poor and marginalized segments, poor implementation, delay in identification. - **Family structure:** nuclear, joint, poor families, lower caste. - **Biological reasons:** care of pregnant women, low birth weight infants, early marriage, hereditary and undernourished mother. - **Lack of awareness:** illiteracy, malnutrition not considered serious, absence of community participation - **Socio-culture beliefs:** myths, practices. - **Unhygienic environment**: sanitation, water, hygiene, unhygienic food offered. - **Status of women:** female, nutrition of women and adolescents, gender equities, poor women care, poor women health.   ***Core theme 1: Multitasking, Time-Constrained Mother with Dwindling Family Support***   - **Working mother:** working women, agriculture work, and MNREGA laborer. - **Mother too busy at home:** always busy in domestic work (buffaloes, cooking, cleaning, wood cutting), workload at home. - **Inability to prepare good food** - **Inability to give good care** - **Nuclear families**: living alone, no support from husband. - **Living in joint family:** with no support from elders and other family members. - **Multiple children** - **Mother health condition and behavior:** fever, micronutrient deficiencies, mentally disturbed, irritated behavior. - **Husband behavior not good /alcoholic** - **Carelessness :**lazy attitude, not fond of children, irresponsible, preference to work , more time in farms, less attention on food. - **Accessibility:** hospital far away from home, medicines not available.   ***Core theme 2: Fragile Food Security/Seasonal Food Paucity***  **Food Accessibility**   - Overall shortage of food. - Household having shortage of cereals, pulses, ghee (at same time of the year). - Preference given to children in case of shortage*.* - Shortage of milk. - Financial Reasons for fragile food security.   **Milk and its use in families**   - No Milk - Milk diluted before giving to children.   ***Core theme 3: Child Targeted Mother***   - **Traditional fast foods**: halwai food items *(samosa, jelabi, pakoda, bread pakoda).* - **Modern fast foods:** market food (maggie, noodles, kurkure, burger, biscuit, tofee, chips). - **Modern fast drinks:** coke, pepsi, sprite. - **Food supplements:** horlicks, complain, cerelac, protein powder. - **Medicinal supplements:** chawanprash, tonics. - **Homemade special dishes**: ghee, halwa, fish and dry food, kheer, fruits. - Availability of market food. - Affordability (small pouches available in 2- 5 RS). - Children like taste. - Attractive packaging /color.   ***Core theme 4: Rising Non-Food Expenditure***   - Non- food expenditure (medical cost, household items, education, communication, cloths). - Use of alcohol.   ***Core theme 5: Adequacy and Appropriateness: home feeding***   - **No special food items prepared:** takes food from everybody plate, regular food. - **Foods with reduced consistency and energy density:** dal water, rice water, dilutes milk; avoid ghee, sugar, specific food items. - **Readymade market food items:** modern fast food (maggie, noodles, kurkure, burger, biscuit, tofee chips, milk powder, supplements) and traditional snacks from sweet shops *(samosa, jelabi, pakoda, bread pakoda).* - **No exclusive breast feeding** for 6 months - **Introduction of weaning foods**< 6 months or > 12 months - **Lack of awareness** (knowledge, illiteracy). - **Poor feeding practices during illness:** Milk dilution, food withholding, serving less food, food avoided. - **Special taste:** spice decreased or increased, sugar added. - **Food with increased energy density:** halwa, kheer, alooparatha, ghee, biscuit-milk. - **Food with increased nutrient density:** khichadi, dal-rice, roti subji, dal.   ***Reasons for not introducing semi solid food items by 7- 9 months***   - Non Acceptance by child/refuse: unable to accept. - Perception that it is too early to initiate semi solid foods. - Adverse effect on child health. - Do not have money to buy.   ***Core theme 6: Delayed Recognition of Under-nutrition and Delayed Care Seeking***   - **Physical appearance:** irritability, crying, sunken eyes. - Less appetite, less feeding, remains sick, frequent illness. - Anthropometry - **Lack of knowledge to** identify. - Recognize when symptoms become/visible/severe under-nutrition. - **Carelessness:** busy family members/ socio cultural beliefs. - Delay in health care /limited resources for medical help. - Socio cultural beliefs.   ***Core theme 7: Inadequate Responsiveness of Integrated Child Development Scheme (ICDS) and Health Care***  **Reasons for non-utilization of Anganwadi Center**   - Poor quality of services and environment at AWCs:lack of trust, confidence, poor quality of services, bad behavior. - Rich and well to do families do not attend only poor, scheduled tribes, scheduled cast, and other backward class families. - Busy mothers, no body to accompany child, migration. - Distance, access. - Lack of awareness of services and benefits of services. - Children going to regular school. - Small child.   **Barriers for utilizing public health services for under five years of children**   - Lack of trust and confidence, poor quality of services. - Poor referral. - Busy mother /unsuitable timing. - Accessibility: inconvenient location of health facility. - Poor awareness of services. - Perceived priority of child's health problems. - Society barriers, class, caste, gender. |
